# Severe hypertriglyceridaemia with length-related small fibre sensory neuropathy as a complication of previous gestational diabetes mellitus

**DOI:** 10.1530/EDM-25-0060

**Published:** 2025-07-22

**Authors:** Fatima Iqbal, Daniel Lim, Ruby Chang, Akhil Gupta, Jeff Ahn, Nimalie Perera

**Affiliations:** ^1^ Department of General Medicine and Endocrinology, Ryde Hospital, Eastwood, New South Wales, Australia; ^2^ College of Medicine, Alfaisal University, Riyadh, Saudi Arabia; ^3^ Department of General Medicine, Ryde Hospital, Eastwood, New South Wales, Australia; ^4^ School of Medicine, University of Newcastle, Newcastle, New South Wales, Australia; ^5^ Department of Endocrinology, Ryde Hospital, Eastwood, New South Wales, Australia; ^6^ Western Sydney University, Blacktown Clinical School and Macarthur Clinical School, Sydney, New South Wales, Australia; ^7^ Department of Diabetes and Endocrinology, Blacktown Hospital, Blacktown, New South Wales, Australia; ^8^ Macquarie Medical School, Faculty of Medicine, Health and Human Sciences, Macquarie University, Sydney, New South Wales, Australia; ^9^ Department of Endocrinology, Royal Prince Alfred Hospital, Sydney, New South Wales, Australia; ^10^ Department of Chemical Pathology, NSW Health Pathology, Royal Prince Alfred Hospital, Camperdown, New South Wales, Australia; ^11^ Sydney Medical School and Charles Perkins Centre, University of Sydney, Sydney, New South Wales, Australia

**Keywords:** hypertriglyceridaemia, small-fibre neuropathy, gestational diabetes mellitus

## Abstract

**Summary:**

Gestational diabetes mellitus (GDM) is a known risk factor for dyslipidaemias. Insulin resistance and the associated dyslipidaemia, particularly hypertriglyceridaemia, have been less frequently linked to peripheral nerve dysfunction, including small fibre sensory neuropathy. The relationship between metabolic disturbances, such as hypertriglyceridaemia, and neuropathy warrants further exploration and has gained increasing recognition in recent studies. This case highlights the potential neurological consequences of lipid abnormalities in women with a history of GDM. A 38-year-old woman presented to an endocrinologist with a 4-week history of paraesthesias and incidental findings of significantly elevated triglycerides (78.4 mmol/L) and total cholesterol (14.7 mmol/L). Initially, numbness began in her left first toe, spreading to other toes on the left foot, and then to the right foot, accompanied by hyperalgesia in fifth fingers bilaterally. She had no history of trauma or back injuries. Her medical history included insulin-dependent GDM and HELLP syndrome 4 years prior, endometriosis, and adenomyosis. With persistently high lipid levels (cholesterol: 12.1 mmol/L; triglycerides: 18.5 mmol/L), she was admitted to ICU for urgent lipid-lowering treatment but experienced hypoglycaemia on an insulin–dextrose infusion. Repeat triglycerides the next day were 13.1 mmol/L. A neurologist diagnosed her with small fibre sensory neuropathy secondary to hypertriglyceridaemia. Treatment with fenofibrate, high-dose fish oil, and a low-fat, low-carbohydrate diet was initiated with outpatient endocrinologist follow-up. Hypertriglyceridaemia is a significant health concern, potentially leading to severe complications such as peripheral neuropathy. Early intervention to optimise lipid levels is essential to prevent adverse outcomes.

**Learning points:**

- GDM is known to be a risk factor for dyslipidaemias.
- Hypertriglyceridaemia can contribute to small fibre sensory neuropathy via mechanisms including microvascular ischaemia, oxidative stress, and inflammation affecting peripheral nerves.
- Diagnosis requires clinical correlation with lipid profiles and neurological findings, and exclusion of other causes through targeted investigations such as nerve conduction studies and autoimmune screening.
- Early recognition of hypertriglyceridaemia is essential to prevent complications such as neuropathy. Acute management may involve insulin–dextrose infusion in cases of severe elevation, while long-term treatment includes fibrates, omega-3 fatty acids, and dietary modifications.

## Background

Dyslipidaemia is characterised by abnormal lipid levels and is a major risk factor for various conditions, including coronary artery disease, stroke, pancreatitis, and, in rare cases, neuropathy. Dyslipidaemia can be inherited or acquired, with familial hypertriglyceridaemia having an estimated prevalence of 1 in 500 individuals (1). Secondary dyslipidaemia is more commonly associated with underlying conditions such as gestational diabetes mellitus (GDM) and metabolic syndrome, and its relationship to peripheral neuropathy, though increasingly recognised, is still understudied (1, 2).

The connection between hypertriglyceridaemia and neuropathy is particularly significant as individuals with metabolic syndrome or a history of GDM may experience worsened lipid profiles postpartum, predisposing them to neuropathic complications (3).

## Case presentation

A 38-year-old Caucasian woman was referred to an endocrinologist for evaluation of hypertriglyceridaemia, incidentally identified during investigations for a 4-week history of paraesthesia. Initial symptoms included numbness in the left first toe, which spread to the left foot and later the right foot. Two weeks later, she developed hyperalgesia in bilateral fifth fingers. There was no history of trauma, fevers, back injuries, or significant weight changes.

Her medical history included both insulin-dependent GDM and HELLP syndrome during her first pregnancy 4 years prior and endometriosis with adenomyosis. She had no family history of diabetes, dyslipidaemia, or relevant systemic disease.

## Investigation

**Figure 1:**
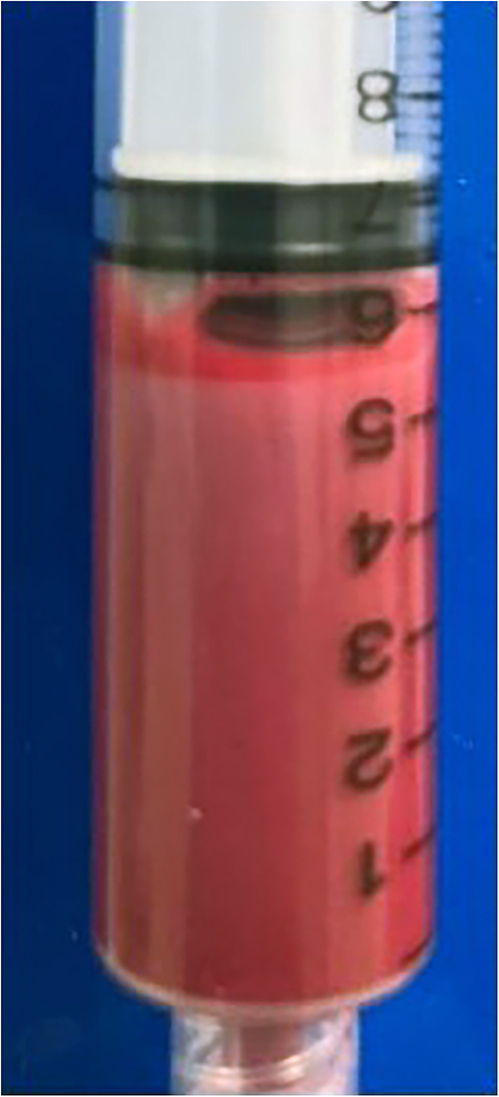
Lipaemic blood sample.

Outpatient blood tests showed triglycerides of 78.4 mmol/L and total cholesterol of 14.7 mmol/L, prompting her admission to a local hospital. Additional blood tests at the time of admission showed a lipaemic sample (Fig. 1), which included HbA1c 5.5%, fasting glucose 3.6 mmol/L, B12 302 pmol/L, folate 24.9 nmol/L, and thyroid stimulating hormone (TSH) 1.83 mIU/L, along with total cholesterol of 12.1 mmol/L and triglycerides of 18.5 mmol/L. HDL was 0.6 mmol/L, and her lipase level was normal (62 U/L), with no symptoms of pancreatitis and no cutaneous findings. There was no medical intervention between the outpatient and hospital blood tests. The marked reduction in triglyceride levels is likely due to factors such as variation in dietary intake before testing and potential laboratory variability. There was no prior lipid profile available for comparison.

Her height was 160 cm and she weighed 67 kg, corresponding to a body mass index (BMI) of 26.2 kg/m^2^.

Neurological examination revealed intact muscle power, normal cranial nerve function, and preserved reflexes, but impaired sensation to pinprick and soft touch. The neurologist diagnosed likely small fibre sensory neuropathy secondary to hypertriglyceridaemia.

## Treatment

The patient was admitted to the ICU for lipid-lowering treatment and started on an insulin–dextrose infusion following local pancreatitis protocols, which was precautionary due to the high risk of developing pancreatitis, with 10% dextrose at 70 mL/hour and Actrapid insulin at 7 units/hour.

Due to ongoing hypoglycaemia despite adjustments – increasing dextrose to 100 mL/hour and reducing insulin to 5 units/hour – the insulin infusion was discontinued after 12 h. Subsequently, the patient was started on fenofibrate and a high dose of generic fish oil.

## Outcome and follow-up

Following insulin–dextrose infusion cessation, repeat triglyceride was 13.1 mmol/L, with total cholesterol 12.1 mmol/L. The patient was discharged on fenofibrate 145 mg daily, fish oil 8 g daily (delivers around 2–3 g of EPA/DHA depending on the formulation), and a low-fat, low-carbohydrate diet with advice to follow-up with her endocrinologist for ongoing monitoring and outpatient referral for genetic testing and nerve conduction studies.

**Figure 2:**
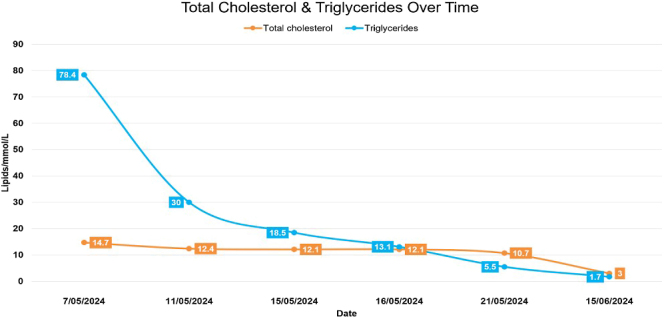
Levels of total cholesterol and triglycerides over time.

The outpatient endocrinologist review one month post-discharge showed a significantly improved lipid profile with total cholesterol 3.0 mmol/L, triglycerides 1.7 mmol/L, HDL 1.0 mmol/L, LDL 1.2 mmol/L, and non-HDL cholesterol 2.0 mmol/L (Fig. 2). Given the rapid normalisation of triglyceride levels with therapy – suggesting a secondary rather than a primary genetic cause – the decision was made not to pursue genetic testing.

Outpatient neurologist review five months post-discharge with nerve conduction studies revealed a mild reduction in the amplitude of plantar responses, suggestive of early length-dependent neuropathy. MRI spine did not reveal significant spinal stenosis. In addition, outpatient investigations including antinuclear antibodies, anti-neutrophil cytoplasmic antibodies, complement levels, extractable nuclear antigen antibodies, cyclic citrullinated antibodies, lupus antibodies, erythrocyte sedimentation rate, neuronal antibodies, coeliac antibodies, serum-free light chains, and serum and urine electrophoresis were performed, all within reference limits.

## Discussion

Hypertriglyceridaemia, characterised by elevated triglyceride levels, is a well-established risk factor for peripheral neuropathy. This condition is associated with symptoms such as pain, numbness, and tingling, particularly in the extremities. The pathophysiology involves microvascular damage, nerve ischaemia, oxidative stress, and inflammation. Elevated triglycerides impair blood flow to small vessels that supply peripheral nerves, causing ischaemia and subsequent nerve injury (1, 4).

The link between hypertriglyceridaemia and neuropathy is more prominent in individuals with metabolic syndrome. Studies suggest that approximately 25–30% of the global population suffers from metabolic syndrome, and the prevalence of peripheral neuropathy among these individuals is high (2, 5, 6). Women with a history of GDM are at increased long-term risk of dyslipidaemia and metabolic syndrome, even in the absence of progression to type 2 diabetes. Longitudinal studies have demonstrated sustained elevations in triglycerides and other lipid abnormalities years after pregnancy, underscoring the lasting metabolic consequences of GDM (7). In addition, studies indicate that 40–50% of women with GDM progress to type 2 diabetes, which further increases their risk of lipid abnormalities and neuropathy (3).

The development of neuropathy in hypertriglyceridaemia involves several interrelated mechanisms. Elevated triglyceride levels can lead to microvascular damage by impairing blood flow to the small blood vessels that supply peripheral nerves, resulting in nerve ischaemia. In addition, increased oxidative stress, characterised by the excessive production of free radicals, contributes to direct damage of nerve tissue. Inflammation is another key mechanism, with elevated triglycerides exacerbating inflammatory responses that further injure peripheral nerves. These pathological processes are commonly observed in individuals with metabolic syndrome – a condition marked by obesity, insulin resistance, hypertension, and dyslipidaemia, including hypertriglyceridaemia (4, 8).

In cases where hypertriglyceridaemia is suspected to have a genetic basis, genetic testing can be a valuable diagnostic tool. Current guidelines recommend considering genetic testing in individuals with triglyceride elevations of uncertain cause, early-onset disease, a significant positive family history of dyslipidaemia or early cardiovascular disease, or a lack of response to conventional therapy. However, the cost of genetic testing – currently non-rebatable under the Australian government healthcare program – can be significant. The targeted gene testing utilises next-generation sequencing to detect single nucleotide and copy number variants in 13 genes associated with primary hypertriglyceridaemia and related conditions: *APOA5, APOC2, APOE, APOB, CREB3L3, GPD1, GPIHBP1, LCAT, LIPA, LIPC, LMF1, LPL,* and *LRP6* (9). The association between hypertriglyceridaemia and peripheral neuropathy underscores the importance of early recognition and management of lipid abnormalities, especially in high-risk populations such as those with metabolic syndrome and a history of gestational diabetes.

The initial treatment of severe hypertriglyceridaemia typically involves rapid lipid reduction, often through insulin–dextrose infusions or plasmapheresis, in cases with impending pancreatitis. Insulin facilitates the clearance of triglyceride-rich lipoproteins by enhancing lipoprotein lipase activity, thereby accelerating the breakdown of very-low-density lipoprotein (VLDL) and chylomicrons. While insulin is effective, fasting alone can also lower triglyceride levels and may be sufficient in patients without pancreatitis. This case highlights the importance of individualised risk–benefit consideration when initiating insulin infusions outside the setting of pancreatitis, particularly to avoid complications such as hypoglycaemia (1).

Long-term management focuses on addressing underlying metabolic risk factors. Lifestyle interventions, particularly dietary modifications (e.g. low-fat, high-omega-3 diet) and regular physical activity, are essential. Pharmacotherapy options include statins, fibrates, and omega-3 fatty acids. Statins primarily target LDL cholesterol but can also reduce triglyceride levels by approximately 10–30%. Fibrates are particularly effective at lowering triglyceride levels – typically achieving reductions of 30–50% – and improve the catabolism of VLDL and chylomicrons through activation of peroxisome proliferator-activated receptor alpha (PPAR-α). Omega-3 fatty acids, known for their anti-inflammatory effects, can reduce triglyceride levels by 20–30% at doses of 2–4 g per day of EPA and/or DHA and also mitigate oxidative stress that contributes to nerve damage (1, 2, 8).

Hypertriglyceridaemia presents significant health risks, including peripheral neuropathy, which can be prevented with early detection and treatment. Optimisation of lipid levels is critical, particularly in high-risk populations such as those with a history of GDM or metabolic syndrome. Genetic testing should be considered when hereditary lipid disorders are suspected, and multidisciplinary care is essential for optimal management.

## Declaration of interest

The authors declare that there is no conflict of interest that could be perceived as prejudicing the impartiality of the work reported.

## Funding

This work did not receive any specific grant from any funding agency in the public, commercial, or not-for-profit sector.

## Patient consent

Written informed consent for publication of their clinical details and/or clinical images was obtained from the patient.

## Author contribution statement

The patient was admitted under the care of JA. FI and DL were part of JA’s team and contributed to the management of the patient. AG, RC, and NP were on the consulting team providing advice to the patient’s care. All authors were involved in writing the manuscript and approved the final draft.
